# Exosomes secreted from induced pluripotent stem cell ameliorate the lipopolysaccharide induced neuroinflammatory response via lncRNA‐0949

**DOI:** 10.1002/iid3.1155

**Published:** 2024-03-27

**Authors:** Lixiu Ma, Ce Xiao, Zhizhe Zhang, Yi‐an Zhan

**Affiliations:** ^1^ Department of Respiratory and Critical Care Medicine the First Affiliated Hospital of Nanchang University Nanchang Jian People's Republic of China; ^2^ Department of Respiratory and Critical Care Medicine Nanchang Jiangxi Province People's Republic of China

**Keywords:** exosome, induced pluripotent stem cell, lncRNA, neuroinflammation

## Abstract

**Purpose:**

To study the effect of exosomes derived from the induced pluripotent stem cells (iPSCs) in the neuroinflammatory response of microglia caused by lipopolysaccharide (LPS) and reveal the potential underlying mechanism.

**Methods:**

A permanent microglia cell line HMO6 was activated by LPS. The features of exosomes were analyzed by nano flow cytometry, Western blot and transmission electron microscope. The RNA‐seq was used to analyze the difference of noncoding RNA profiles between iPSC‐Exos and HMO6 derived exosomes and proved that long no‐coding RNA (lncRNA‐0949) was highly expressed in the iPSC‐Exos. Activated HMO6 cells were cocultured with iPSC‐Exos in which lncRNA‐0949 was overexpressed, knocked down or normally expressed. Quantitative real‐time polymerase chain reaction (RT‐qPCR), Enzyme‐Linked Immunosorbent Assay and Western blot assay were adopted to analyze RNA and protein expression of inflammatory factors in HMO6 cells.

**Results:**

The oxidative stress and inflammatory response of microglia were significantly attenuated with the iPSC derived exosomes treatment. LncRNA‐0949 was effectively delivered into the HMO6 cells through the iPSC‐Exos, which largely alleviated the production of malondialdehyde, IL‐6, IL‐1β and TNF‐α in HMO6 cells. Overexpression of lncRNA‐0949 could enhance the anti‐inflammatory effect of the iPSC‐Exos, and knock‐down of lncRNA‐0949 impaired this availability.

**Conclusion:**

According to our results, lncRNA‐0949 enriched exosomes from iPSC could potentially be used as a therapeutic strategy to prevent/treat neuroinflammatory diseases.

## 1 INTRODUCTION

In cases of acute neuropathology, microglia are the resident macrophages of the central nervous system (CNS) that are rapidly activated.^1^ They are key cellular mediators in neurodegeneration and neuroinflammation.^2^ Microglia populations are heterogeneous, and microenvironments play a role in influencing microglia phenotypes.^3^ They are able to distinguish between functionally different phenotypes, M1 and M2.^4^ It is believed that M1 microglia are proinflammatory cells, activated by toll‐like receptors (TLRs) or interferon‐gamma, and release proinflammatory cytokines and reactive oxygen species which can damage tissues.^5^ Microglia become active when they are transformed into phagocytes, releasing potentially cytotoxic substances such as oxygen free radicals, nitric oxide, proteases and proinflammatory cytokines.^6^, ^7^ According to recent studies, activation of microglia precedes degeneration of neurons and glia in neurological disorders such as Alzheimer's disease (AD), Parkinson's disease, and multiple sclerosis.^8^, ^9^, ^10^

The field of stem cells has made remarkable progress since Yamanaka discovered induced pluripotent stem cells (iPSCs) in 2006.^11^ Somatic reprogramming avoids the challenges of immune rejection and ethical concerns surrounding human embryonic stem cells.^12^ iPSC technology constantly improves efficiency of vitro models construction and drug discovery.^13^, ^14^, ^15^, ^16^ However, significant challenges remain in transforming iPSC biology into a clinical reality, such as potential tumorigenicity.^17^ Exosomes are subsets of extracellular vesicles (EVs), which originate from intracellular compartments and are mainly regulated by endosomal sorting complex required for transport (ESCRT).^18^ Stem cell‐derived exosomes play a beneficial role in the stimulation or signaling pathways of endogenous repair in human CNS diseases, particularly through microRNAs (miRNAs), long no‐coding RNAs (lncRNAs), small molecules and proteins that mediate pro‐angiogenesis, proliferation, antiapoptotic and anti‐inflammatory effects.^19^, ^20^, ^21^, ^22^ Studies like these shed light on precision medicine's future, in which the application of exosomes derived from somatic cells provides a new personalized therapeutic approach.

In this study, we aimed to evaluate the effect of iPSC derived exosomes (iPSC‐Exos) on the lipopolysaccharide (LPS) induced neuroinflammation and reveal the potential underlying mechanism.

## 2 MATERIAL AND METHODS

### 2.1 Cell culture

In this study, we used human iPSC line and human permanent microglial cell line HMO6, both of which were obtained from Genelily BioTech Co., Ltd. Human skin fibroblasts were cultured with sendai virus expressing transcriptional factors Oct4, Sox2, cMyc and Klf4 through using a CytoTuneTM‐iPS 2.0 Sendai Reprogramming Kit (ThermoFisher Scientific). Cells were replated in Geltrex‐coated petri dishes 3 days after infection. The cells were cultured in Essential 8 medium (ThermoFisher Scientific) and passed every 3 days. A humidified atmosphere containing 5% CO_2_ and DMEM supplemented with 10% fetal bovine serum and penicillin/streptomycin at 37°C was used to culture HMO6 cell line.

### 2.2 Isolation and characterization of exosomes

Replace human pluripotent stem cell cultures with fresh Essential 8 culture solution every day. The waste medium was collected daily from Day 2 until the end of culture and filtered with a 0.45 μm syringe filter to remove cellular debris. Exosomes were isolated from conditioned media using Exoquick‐TC (System Biosciences) according to manufacturer's instructions. In brief, the conditioned medium was incubated with exosome precipitate at 4°C for 12 h and then centrifuged at 1500 *g* for 30 min. After the supernatant was discarded, the supernatant was suspended again with 50 μL phosphate buffered saline (PBS, pH = 7.2), and the collected iPSC‐Exos were stored at −80°C for later use. The size distribution and concentration of iPSC‐Exos were analyzed by the Flow NanoAnalyzer (NanoFCM Inc.) according to the manuscript.^23^ Briefly, iPSC‐Exos were incubated fluorescent primary antibodies at room temperature for 30 min, protected from light. Then stained iPSC‐Exos were washed with PBS and centrifuged at 10,000 *g* and 4°C for 1 h to remove antibody in excess. The 200 nm silica QC beads (2.01 × 10^10^ particles/mL) were to quality control before sample analyzing. iPSC‐Exos were diluted with PBS to appropriate concentration (2.98 × 10^8^ particles/mL). The sample was measured with 19.3 nL/min. The morphology of iPSC‐Exos was observed by transmission electron microscopy (TEM) using a JEM‐1010 electron microscope (JEOL). iPSC‐Exos was adsorbed on a formvar/carbon‐coated grid for 10 min and then fixed with 2% paraformaldehyde (PFA). After negative staining with 2% uranyl acetate for 10 min, iPSC‐Exos was observed by TEM at 60 kV. Zeta potential was measured using Zetasizer Nano ZS (Malvern Panalytical). All experiments kept camera level, threshold and focal length unchanged. The expression levels of exosome markers CD63, TSG101 and Alix1 were assayed by Western blot.

### 2.3 Quantitative real‐time polymerase chain reaction (RT‐qPCR)

Cells were lysed with TRIzol reagent for total RNA extraction and cDNA was synthesized using a one‐step RT‐PCR kit (ThermoFisher Scientific). RT‐qPCR was performed using ABI Vii7 system (Applied Biosystems). GAPDH is the housekeeping gene. The $2^{‐△△C_{T}}$ cycle threshold method^24^ was used to calculate relative gene expression levels. Primers used for RT‐qPCR analysis were listed at Table 1.

**Table 1:** Primers used for RT‐qPCR analysis.

Gene	Forward primer (5′‐3′)	Reverse primer (5′‐3′)
MIP2	CTGCCAGTGCTTGCAGACCC	TTAACCATGGGCGATGCGGG
TNF‐α	CAAAGTAGACCTGCCCAGAC	GACCTCTCTCTAATCAGCCC
IL‐1β	AAAAGCTTGGTGATGTCTGG	TTTCAACACGCAGGACAGG
IL‐6	GTGTGAAAGCAGCAAAGAGGC	CTGGAGGTACTCTAGGTATAC
GAPDH	TGACTTCAACAGCGACACCCA	CACCCTGTTGCTGTAGCCAAA

Abbreviations: MIP2, microphage inflammatory protein‐2; RT‐qPCR, quantitative real‐time polymerase chain reaction.

### 2.4 Overexpression and knock‐down of lncRNA‐0949 in iPSCs

To obtain cell line over‐expressing lncRNA‐0949, lncRNA‐0949 cDNA was amplified and subcloned into the pLVX‐IRES‐Puro lentiviral vector (Genelily). Recombinant lentiviruses containing the lncRNA‐0949 gene (Lv‐lncRNA‐0949) were obtained from Genelily BioTech Co., Ltd. Stable cells were obtained by treating iPSC cells with 2 mg/mL puromycin for 2 weeks after infection. To obtain cell stable knock‐down lncRNA‐0949, the lentivirus vector containing lncRNA‐0949 shRNA (Lv‐lncRNA‐0949‐shRNA) was amplified and cloned. Infected iPSC cells were selected by treating them with puromycin (2 mg/mL) for 2 weeks after infection with concentrated virus.

### 2.5 Internalization of iPSC‐Exos into HMO6 cells

The iPSC‐Exos were labeled with the PKH67 dye (MedChemExpress) for 4 min. Bovine Serum Albumin was used to terminate staining. The iPSC‐Exos were isolated by using Exoquick‐TC to remove excess dye. HMO6 cells cocultured with iPSC‐Exos labeled with PKH67 for 12 h. After fixation with 4% PFA for 30 min, the samples were observed under a fluorescence microscope (Leika).

### 2.6 Malondialdehyde (MDA) analysis

To measure MDA concentration, the sample was reflowed with a solution of HCl and TBA. 2 mL was added into the supernatant of 1 mL HMO6 cell culture medium, heated in 50°C water bath for 40 min, and TBA was dissolved. After cooling to room temperature, centrifuge at 1000 RPM for 10 min, absorbance was read at 535 nm, and MDA concentration was calculated by *C* (M) = *A*/1.65 × 10^5^, where *C* is concentration and *A* is absorbance.

### 2.7 Enzyme‐linked immunosorbent assay (ELISA)

Levels of Macrophage inflammatory protein‐2 (MIP2), tumor necrosis factor α (TNF‐α), interleukin‐1β (IL‐1β) in HMO6 cell culture medium were determined using commercially available ELISA kits (eBioscience) according to manufacturer's instructions. In simple terms, take 100 μL supernatant, diluted standard, quality control, and diluted buffer (blank) and place on a precoated plate containing monoclonal antibody for 2 h. Add 100 μL biotin‐labeled antibody and incubate for 1 h. Wash the plate, add 100 μL streptavidin‐HRP conjugate, and incubate in the dark for 30 min. Add 100 μL substrate and incubate for 15 min. Adding stop solution indicates the last step before reading the absorbance (450 nm) on the microplate reader.

### 2.8 Western blot

HMO6 cells or exosomes were lysed with RIPA buffer and total proteins concentration was determined with the BCA kit. 20−50 μg of total protein was loaded into each lane of gel. After electrophoresis, the blot was incubated with primary antibodys over night at 4°C. An ChemiDoc MP Imaging System (Bio‐Rad, CA, USC) was used to visualize Western blot with corresponding horseradish peroxidase‐conjugated secondary antibody. Primary antibodies used were anti‐p‐P38, anti‐P38, anti‐TLR4, anti‐p‐ERK1/2, anti‐ERK1/2, anti‐NF‐κB p65, anti‐Histone H3 and anti‐GAPDH (all from Abcam). GAPDH and Histone H3 as endogenous controls.

### 2.9 Statistical analysis

Data are presented as mean ± SD (mean ± standard deviation) for at least three independent experiments. Statistical analyses have been performed using GraphPad Prism 9 software (GraphPad Software Inc.). One‐way analysis of variance and Student *t* tests were used to compare means between groups. It is statistically significant at *p* < .05.

## 3 RESULTS

### 3.1 Characterization of exosomes derived from human iPSCs

**Figure 1:**
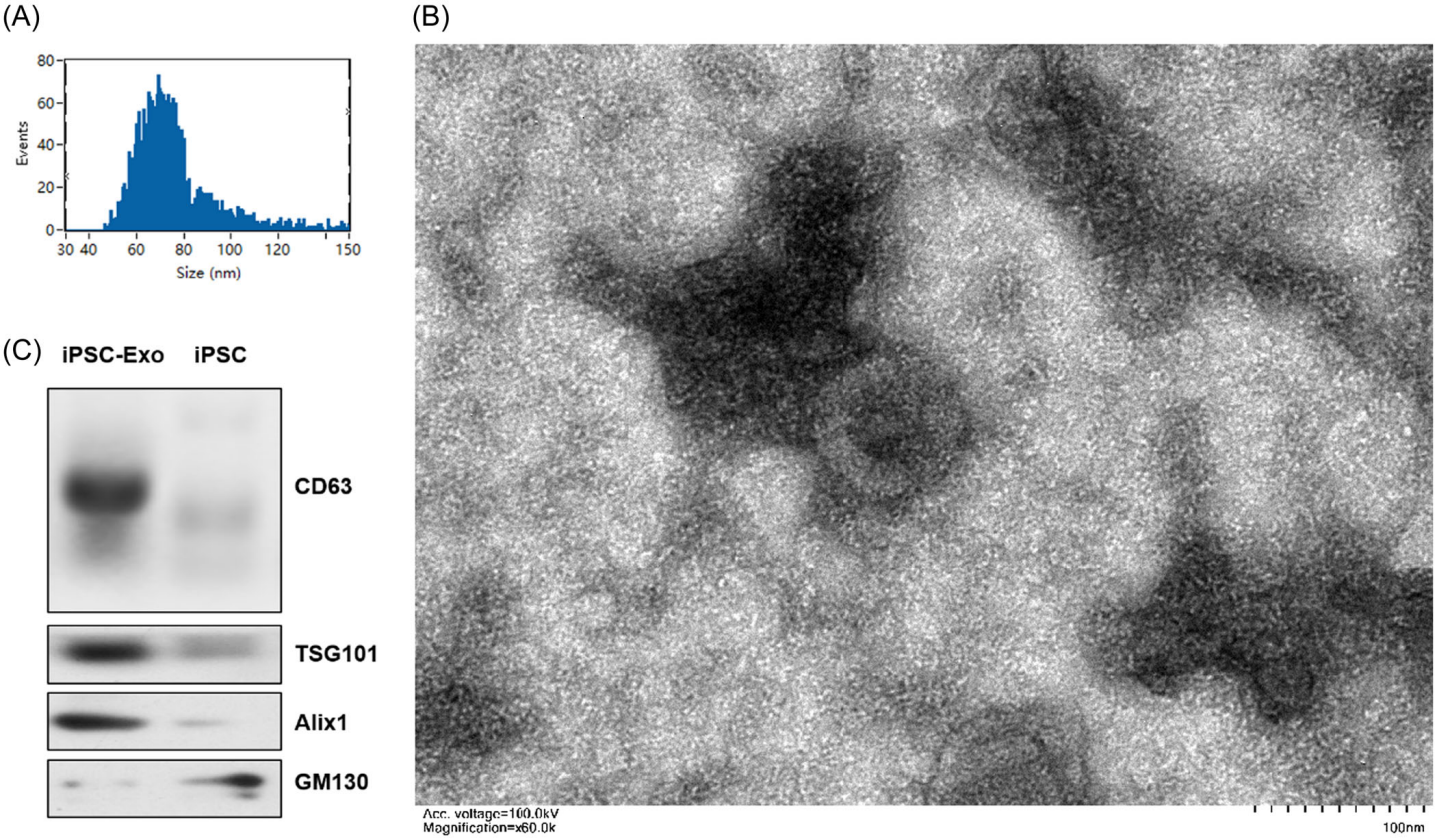
Characterization of exosomes derived from human iPSCs. (A) The nFCM analysis was used to analysis the size distribution of iPSC‐Exos. (B) Transmission electron microscopy (TEM) image showed that iPSC‐Exos had a spherical membrane structure. (C) Immunoblot analysis was used to confirm the presence of expected exosomal marker proteins (CD63, Alix1, and TSG101). GAPDH: Glyceraldehyde 3‐phosphate.

Exosomes were isolated from human iPSCs and characterized in size and morphology. We used Nano flow cytometry (nFCM) to assess the number and size of exosomes. nFCM result showed that the size distribution of iPSC‐Exos had a main peak at ~100 nm, with an average diameter of 76.5 nm (Figure 1A). This result indicated that most of the extracellular vesicles used in this study were exosomes, because other microvesicles are larger in size than exosomes. TEM images showed that iPSC‐Exos were a spherical membrane structure and exosome size was similar to nFCM result (Figure 1B). Western blot analysis was used to confirm the presence of expected exosome marker proteins (CD63, Alix1, and TSG101) and the absence of GM130. (Figure 1C).

#### 3.1.1 Exosomes derived from iPSCs dose‐dependently ameliorated the LPS induced neuroinflammatory response in HMO6 microglia cells

**Figure 2:**
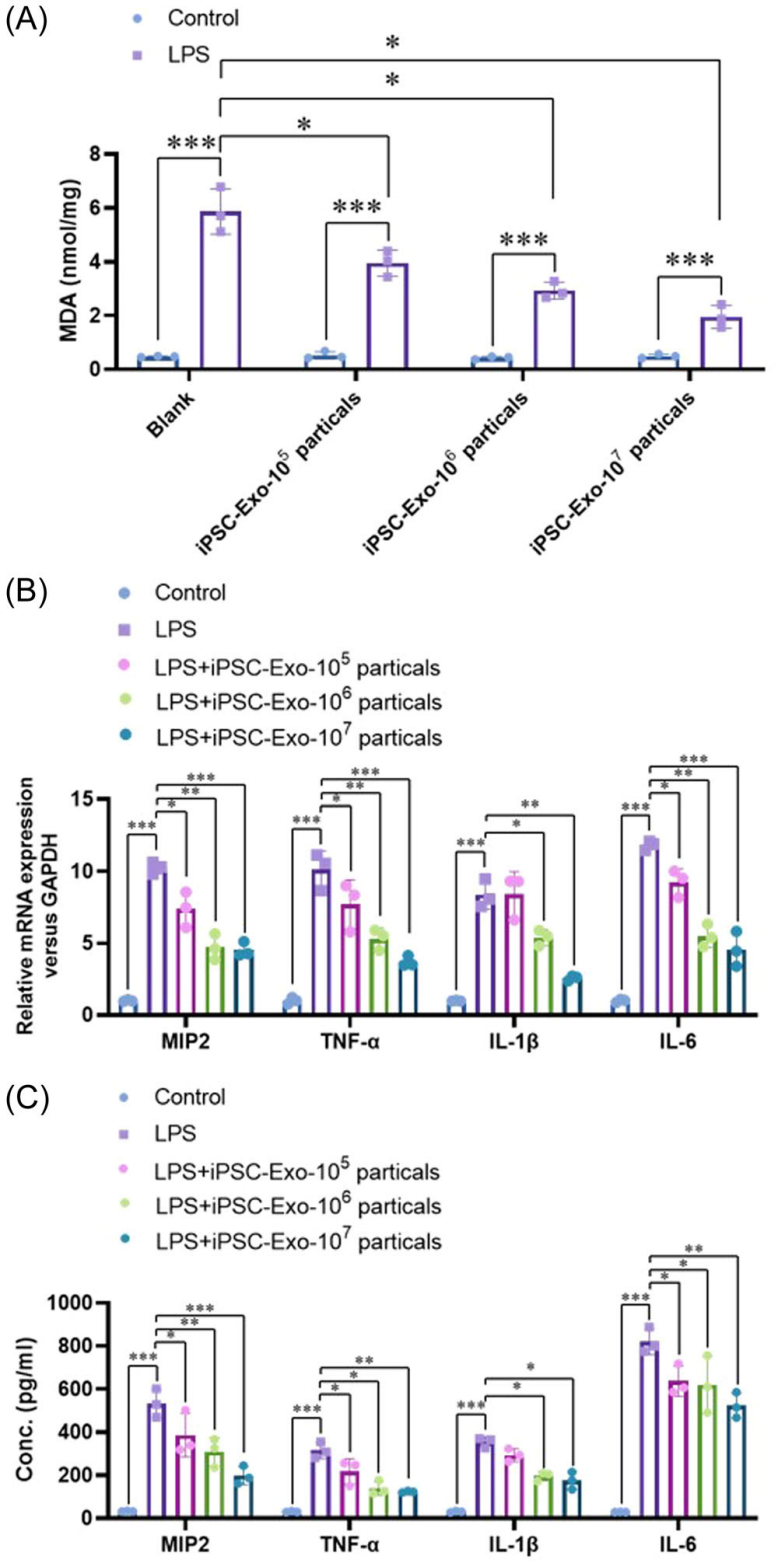
Exosomes derived from iPSCs dose‐dependently ameliorate the LPS induced neuroinflammatory responses in HMO6 microglia cells. (A) The level of MDA concentration in HMO6 cells were detected by Kit. (B) The mRNA expressions of these cytokines (MIP2, TNF‐α, IL‐1β, and IL‐6) were analyzed by real‐time PCR. (C) The protein levels of these cytokines (MIP2, TNF‐α, IL‐1β and IL‐6) were further confirmed by the ELISA analysis. *p < .05, **p < .01, ***p < .001 compared with indicated group.

To identify whether exosomes derived from iPSCs affect the LPS induced inflammation response in microglia cells, we used the permanent microglia cell line HMO6 and induced the neuroinflammatory response with LPS (100 ng/mL). As shown in Figure 2A, only iPSC‐Exos treatment did not alter the level of MDA concentration, whereas LPS treatment significantly elevated the level of MDA concentration in HMO6 cells. Notably, different concentration of iPSC‐Exos dramatically impaired the LPS induced MDA elevation, which suggested a protective role of iPSC‐Exos on the LPS induced oxidative stress in HMO6 cells. We also analyzed the mRNA expression levels of critical inflammation cytokines (MIP2, TNF‐α, IL‐1β, and IL‐6). Consistently, the mRNA expressions of these cytokines were obviously increased by LPS treatment, which were dose‐dependently attenuated via treating iPSC‐Exos (Figure 2B). The protein levels of these cytokines in the were further confirmed by the ELISA analysis (Figure 2C). These results demonstrate that exosomes derived from iPSCs dose‐dependently ameliorate the LPS induced inflammation response in the HMO6 microglia cells.

#### 3.1.2 LncRNA‐0949 was enriched in the iPSC‐Exos and delivered into the HMO6 microglia cells

**Figure 3:**
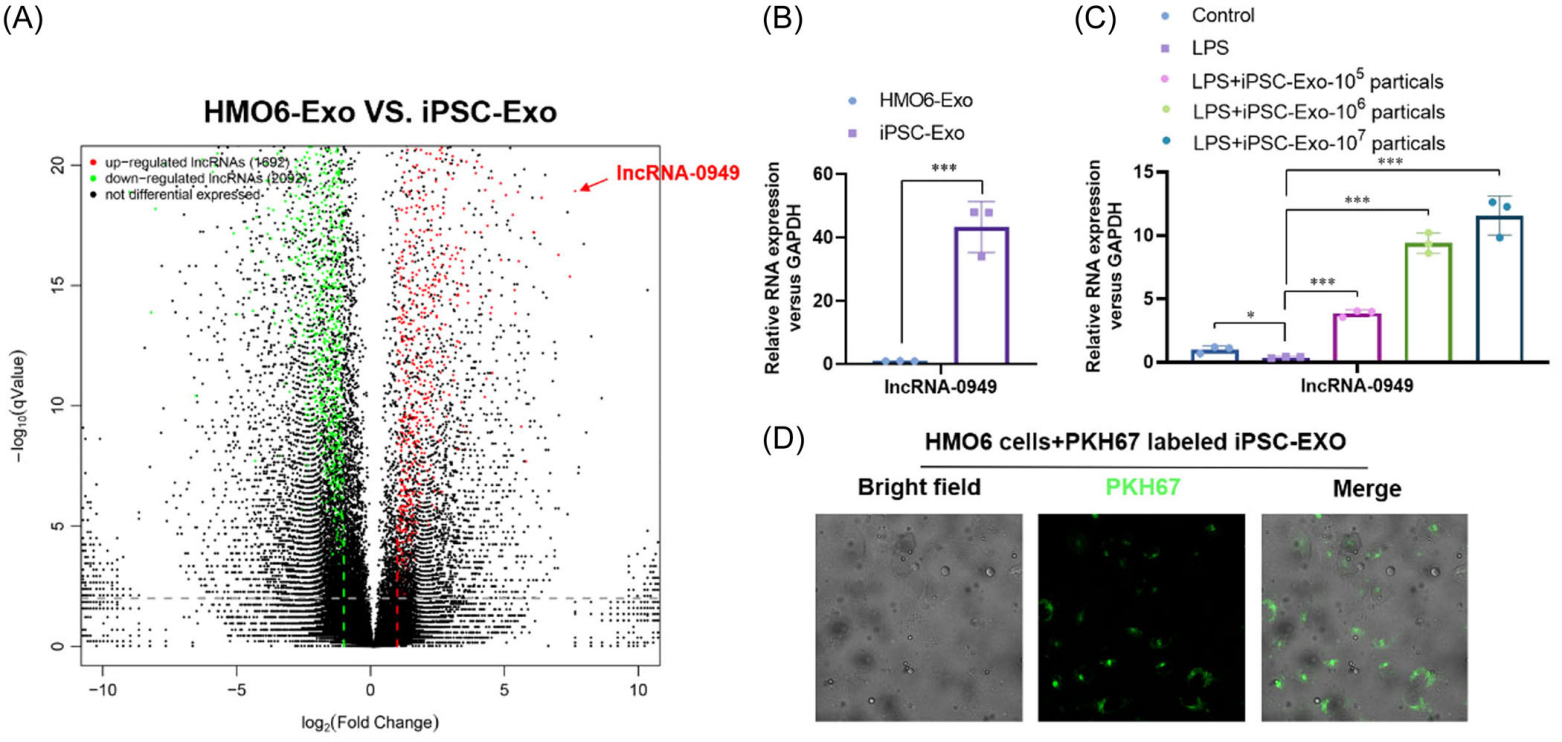
LncRNA‐0949 was enriched in the iPSC‐Exos and delivered into the HMO6 microglia cells. (A) The profiles of noncoding RNA in iPSC‐Exos and compared the differences with HMO6 derived exosomes was analyzed by RNA‐seq. (B) The level of lncRNA‐0949 in iPSC‐Exos was verified by real‐time PCR. (C) The level of lncRNA‐0949 in the iPSC‐Exos treated HMO6 cells was analyzed by real‐time PCR. (D) After PKH67 labeling of iPSC‐Exos, the uptake of iPSC‐Exos by HMO6 cells was detected by fluorescence microscope. *p < .05, **p < .01, ***p < .001 compared with indicated group.

To explore the underlying mechanism of iPSC‐Exos relieving the LPS induced inflammation response in the microglia, we compared noncoding RNA profiles of the iPSC‐Exos and HMO6 derived exosomes. A significant enrichment of lncRNA‐0949 was shown in iPSC‐Exos (Figure 3A) and further verified by real‐time PCR (Figure 3B). Moreover, we also observed a significant increase of lncRNA‐0949 after iPSC‐Exos treated HMO6 cells (Figure 3C). The uptake of iPSC‐Exo by HMO6 cells was detected by fluorescence microscope (Figure 3D). These results suggested that lncRNA‐0949, enriched in the iPSC‐Exos, is delivered into the HMO6 microglia cells and contributes to anti‐inflammatory role of iPSC‐Exo.

#### 3.1.3 The iPSC‐Exos ameliorated the LPS induced neuroinflammatory response via delivering lncRNA‐0949 in HMO6 microglia cells

**Figure 4:**
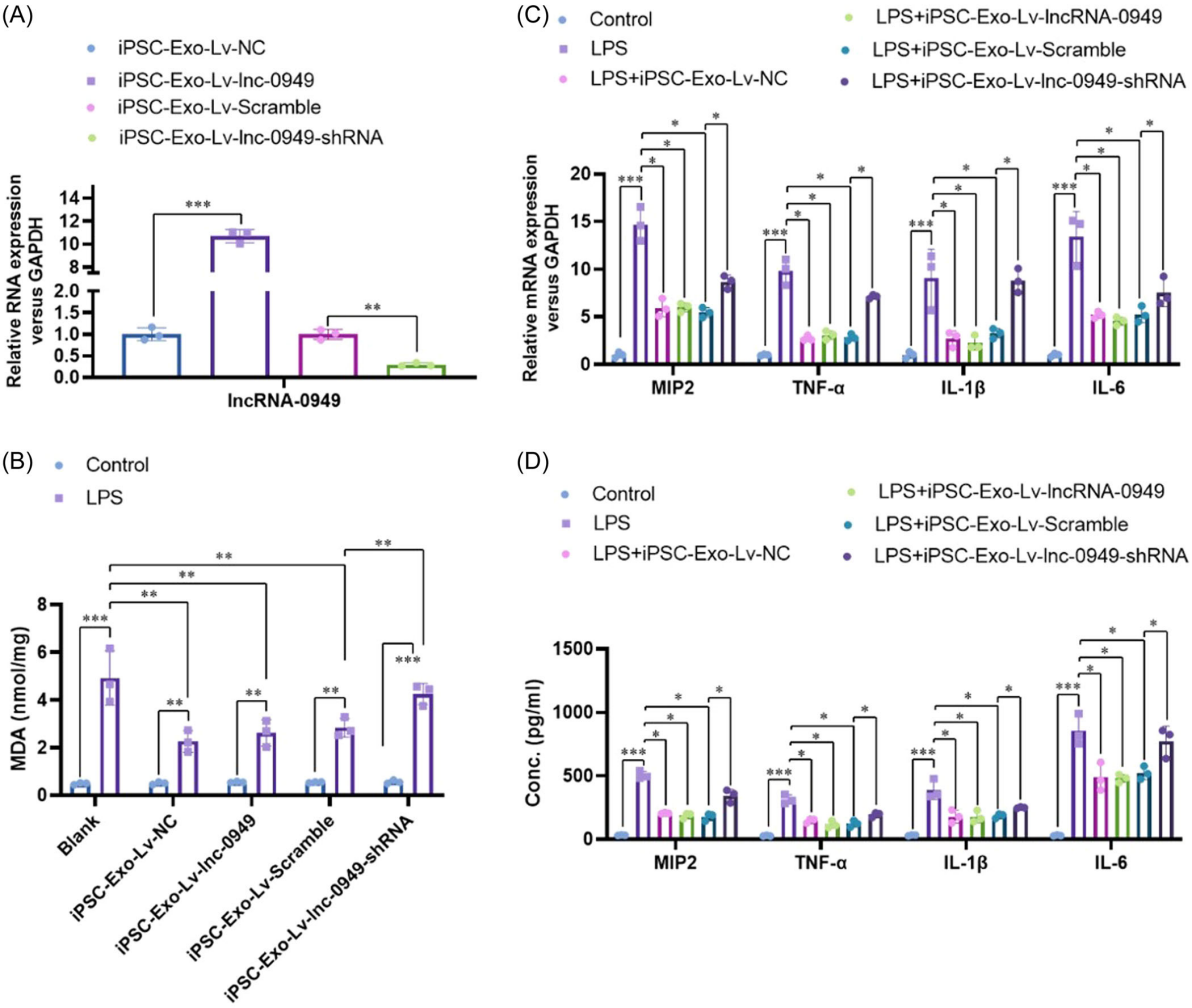
The iPSC‐Exos ameliorate the LPS induced neuroinflammatory responses via delivery lncRNA‐0949 in HMO6 microglia cells. (A) The overexpressed and knockdown of lncRNA‐0949 in iPSC‐Exos were confirmed by real‐time PCR. (B) The level of MDA concentration in HMO6 cells were detected by Kit. (C) The mRNA expressions of these cytokines (MIP2, TNF‐α, IL‐1β, and IL‐6) were analyzed by real‐time PCR. (D) The protein levels of these cytokines (MIP2, TNF‐α, IL‐1β and IL‐6) were further confirmed by the ELISA analysis. *p < .05, **p < .01, ***p < .001 compared with indicated group.

**Figure 5:**
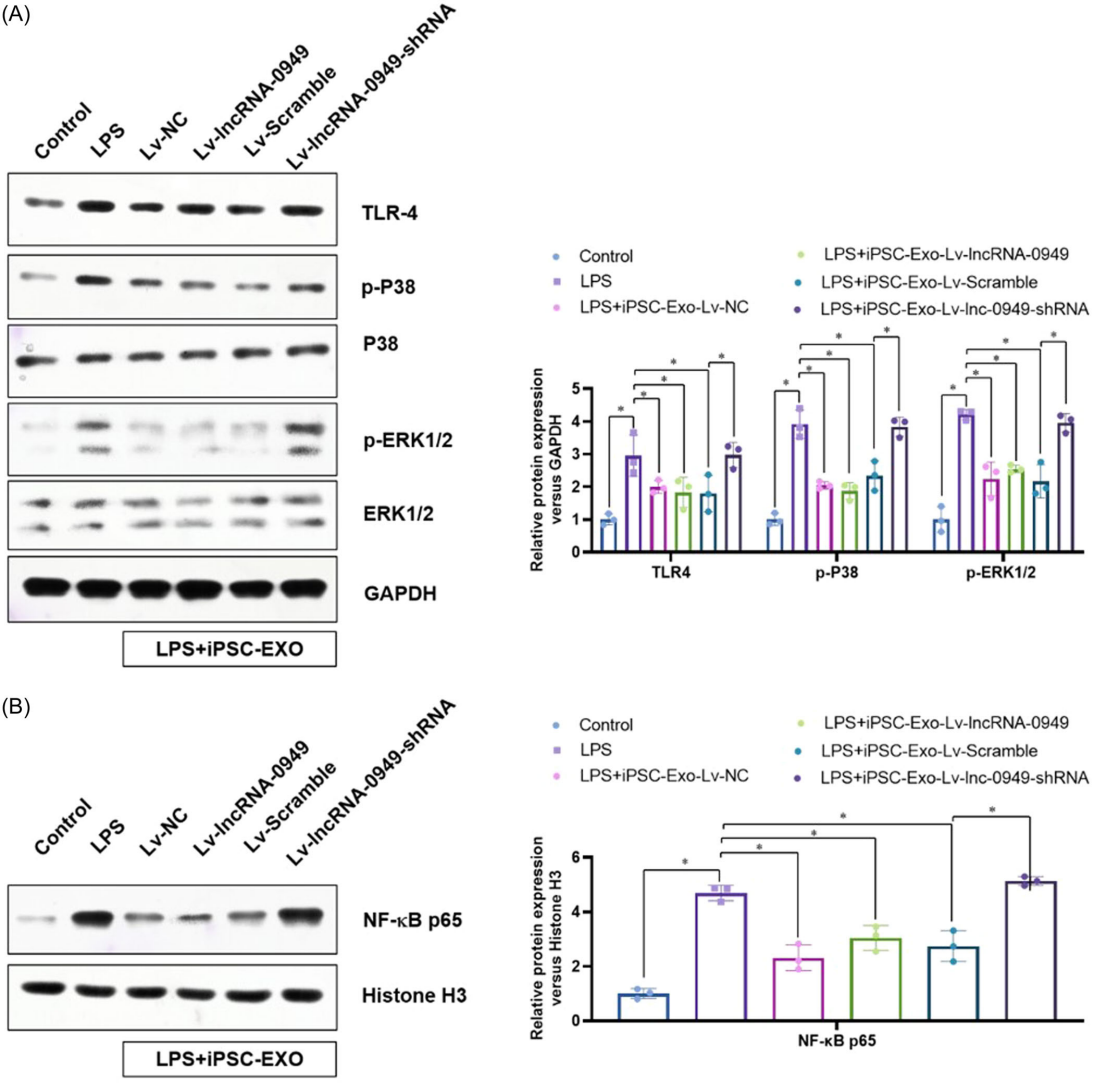
The iPSC‐Exos ameliorate the LPS induced neuroinflammatory signaling via delivery lncRNA‐0949 in HMO6 microglia cells. (A) The LPS activated critical inflammation signaling (including TLR4, phosphorylation of p38 and ERK1/2) were analyzed by Western blot. (B) The nucleus translocation of NF‐κB p65 were analyzed by nuclear component extraction and Western blot analysis. *p < .05, **p < .01, ***p < .001 compared with indicated group.

To understand the critical role of lncRNA‐0949 ameliorating inflammation response in HMO6 microglia cells, we overexpressed and knock‐downed lncRNA‐0949 in iPSC, and isolated their derived exosomes (Figure 4A). These exosomes did not affect the MDA levels in the normal HMO6 cells. After LPS treatment, lncRNA‐0949 overexpressed iPSC‐Exos (iPSC‐Exo‐Lv‐lncRNA‐0949) showed a comparable inhibition of LPS induced MDA with normal control (iPSC‐Exo‐Lv‐NC). However, lncRNA‐0949 knock‐downed iPSC‐Exos (iPSC‐Exo‐Lv‐lncRNA‐0949‐shRNA) showed a limited inhibition of LPS induced MDA (Figure 4B). Similar changes were also observed on the mRNA expression (Figure 4C) and protein (Figure 4D) levels of critical inflammation cytokines (MIP2, TNF‐α, IL‐1β and IL‐6). Furthermore, the LPS activated critical inflammation signaling (including TLR4, phosphorylation of p38 and ERK1/2) (Figure 5A) and nucleus translocation of NF‐κB p65 (Figure 5B) were comparably inhibited by iPSC‐Exo‐Lv‐NC, iPSC‐Exo‐Lv‐lncRNA‐0949 and iPSC‐Exo‐Lv‐Scramble, which were reversed by lncRNA‐0949 knockdown. These data collectively demonstrate that the iPSC‐Exo ameliorates the LPS induced neuroinflammatory response via delivery lncRNA‐0949 in HMO6 cells.

## 4 DISCUSSION

As resident macrophages of the CNS, microglia play a crucial role in innate immunity and are the first line of defense against exogenous toxic substances and proinflammatory response. Microglia are involved in neuroprotection in the normal brain, as phagocytes remove cell debris and damaged neurons.^25^, ^26^, ^27^ Despite this, abnormally activated microglia and astrocytes significantly promote neuroinflammation and neurotoxic response by releasing a range of proinflammatory cytokines and mediators, including IL‐1β, IL‐6, TNF‐α, Prostaglandin‐endoperoxide synthase 2 (COX‐2) and nitric oxide synthase.^28^, ^29^ Neuroinflammation can lead to synaptic degeneration, neuronal cell death, and cognitive dysfunction in neurodegenerative diseases such as Alzheimer's disease.^30^, ^31^, ^32^ Therefore, modulating neuroinflammatory response may be an effective strategy to combat neuroinflammatory and neurodegenerative diseases. LPS, as main cell wall component of gram‐negative bacteria, acts as a strong activator of microglia.^33^ A neurodegenerative model demonstrates that microglial activation by LPS promotes disease progression.^34^

Exosomes, 30−150 nm in diameter, are essentially intracavity vesicles produced by the budding of intracellular multivesicular body.^35^ They participate in cell communication by mediating horizontal transfer of nucleic acids, proteins and lipids. Furthermore, exosomes may be a nontoxic, nonimmunogenic, and natural carriers with good biocompatibility for the delivery of bioactive contents.^36^, ^37^ iPSCs hold great promise in tissue regeneration and anti‐inflammationand avoiding immune rejection.^14^, ^15^ In this study, we generated iPSCs by reprogramming human skin fibroblasts. Subsequently, iPSC‐Exos were isolated and characterized by nFCM, Western blot and TEM. We found that the exosomes derived from iPSCs could inhibit the LPS induced inflammatory response in the permanent microglia cell line HMO6.

Regulatory noncoding RNAs, such as lncRNA, play a key role in life's activities.^38^ The CNS of higher mammals contains a large number of lncRNA, which are likely due to the complex function and structure of brain, and requiring more regulatory RNA to maintain its normal development and function.^2^, ^39^, ^40^ Many studies have shown that lncRNA are involved in neuronal differentiation, brain development and synaptic plasticity.^41^ Recently, lncRNA H19 was enriched in the exosomes from bone marrow mesenchymal stem cells (BMSCs‐Exos) and inhibited LPS induced inflammatory response in the microglia and neuron apoptosis by sponging miR‐29b‐3p.^42^ In this study, we evaluated the noncoding profiles in the exosomes and found that lncRNA‐0949 was enriched in the iPSC‐Exos and delivered into the HMO6 microglia cells. Moreover, we confirmed that the iPSC‐Exos could ameliorate the LPS induced neuroinflammatory response via delivering lncRNA‐0949 in HMO6 microglia cells.

Microglial cells have TLRs such as TLR4 which interacts with LPS.^43^, ^44^ When TLR4 signaling is activated, NF‐κB and/or other transcription factors in the nucleus are affected and proinflammatory cytokines are released.^45^ Herein, we also revealed that the LPS activated inflammation signaling (including TLR4, phosphorylation of p38 and ERK1/2) and nucleus translocation of NF‐κB p65 was comparably inhibited by iPSC‐Exo‐Lv‐NC, iPSC‐Exo‐Lv‐lncRNA‐0949 and iPSC‐Exo‐Lv‐Scramble, which was reversed by lncRNA‐0949 knockdown.

This study has provided valuable insights into the potential therapeutic role of iPSC‐derived exosomes in neuroinflammatory diseases. However, there are some limitations in this research: (1) The study utilized a permanent microglial cell line (HMO6) to induce neuroinflammatory responses. While cell lines can provide preliminary insights, they may not fully recapitulate the complexity of the human brain environment. Future studies should consider using primary microglial cells or animal models to validate the findings; (2) Although the study identified lncRNA‐0949 enriched in iPSC‐derived exosomes regulated NF‐kB pathway to inhibit microglial inflammation, the exact mechanisms remain unclear. Further investigations should be conducted to elucidate the molecular pathways and targets involved; (3) While the study provides important in vitro evidence, the therapeutic potential of iPSC‐derived exosomes and lncRNA‐0949 should be further evaluated in preclinical animal models. Animal studies can help assess the safety, efficacy, and long‐term effects of this therapeutic approach.

## 5 CONCLUSION

In summary, we found an inhibitory role of exosomes derived from iPSCs on the LPS induced neuroinflammatory response in microglia. Moreover, we verified that the exosomes derived from iPSCs delivered lncRNA‐0949 into the HMO6 cells to act this anti‐inflammatory function. Accordingly, this study suggests that iPSCs enriched with lncRNA‐0949 may be useful as a therapy for treating neuroinflammation‐related disorders.

## AUTHOR CONTRIBUTIONS

*Conception and design*: Yi‐an Zhan. *Analyze and process data*: Lixiu Ma, Ce Xiao. *Drafted the manuscript*: Zhizhe Zhang and Lixiu Ma. *Review and editing*: Yi‐an Zhan and Lixiu Ma.

## CONFLICT OF INTEREST STATEMENT

The authors declare no conflict of interest.

## Data Availability

The data that support the findings of this study are available from the corresponding author upon reasonable request.

## References

[1] Borst K , Dumas AA , Prinz M . Microglia: immune and non‐immune functions. Immunity. 2021;54(10):2194‐2208. 34644556 10.1016/j.immuni.2021.09.014

[2] Absinta M , Maric D , Gharagozloo M , et al. A lymphocyte‐microglia‐astrocyte axis in chronic active multiple sclerosis. Nature. 2021;597(7878):709‐714. 34497421 10.1038/s41586-021-03892-7 PMC8719282

[3] Chen Y , Colonna M . Microglia esprit de corps: sharing the burden of eliminating toxic aggregates. Cell. 2021;184(20):5082‐5084. 34597598 10.1016/j.cell.2021.08.033

[4] Croese T , Castellani G , Schwartz M . Immune cell compartmentalization for brain surveillance and protection. Nature Immunol. 2021;22(9):1083‐1092. 34429552 10.1038/s41590-021-00994-2

[5] Lewis S . Microglia prune inhibitory synapses, too. Nat Rev Neurosci. 2021;22(9):517. 34312535 10.1038/s41583-021-00504-1

[6] Pascoal TA , Benedet AL , Ashton NJ , et al. Microglial activation and tau propagate jointly across braak stages. Nature Med. 2021;27(9):1592‐1599. 34446931 10.1038/s41591-021-01456-w

[7] Popova G , Soliman SS , Kim CN , et al. Human microglia states are conserved across experimental models and regulate neural stem cell responses in chimeric organoids. Cell Stem Cell. 2021;28:2153‐2166.e6. 34536354 10.1016/j.stem.2021.08.015 PMC8642295

[8] Wood H . Astrocytic IL‐3 could help microglia protect against alzheimer disease. Nat Rev Neurol. 2021;17(9):525. 10.1038/s41582-021-00546-0 34321636

[9] Wright R . Microglia set the pace for tau spread. Nature Neurosci. 2021;24(10):1342. 10.1038/s41593-021-00931-4 34588697

[10] Breunig M , Merkle J , Wagner M , et al. Modeling plasticity and dysplasia of pancreatic ductal organoids derived from human pluripotent stem cells. Cell Stem Cell. 2021;28(6):1105‐1124.e19. 33915078 10.1016/j.stem.2021.03.005 PMC8461636

[11] Dannenmann B , Klimiankou M , Oswald B , et al. iPSC modeling of stage‐specific leukemogenesis reveals BAALC as a key oncogene in severe congenital neutropenia. Cell Stem Cell. 2021;28(5):906‐922.e6. 33894142 10.1016/j.stem.2021.03.023

[12] Huang WK , Wong SZH , Pather SR , et al. Generation of hypothalamic arcuate organoids from human induced pluripotent stem cells. Cell Stem Cell. 2021;28(9):1657‐1670.e10. 33961804 10.1016/j.stem.2021.04.006 PMC8419002

[13] Manian KV , Galloway CA , Dalvi S , et al. 3D iPSC modeling of the retinal pigment epithelium‐choriocapillaris complex identifies factors involved in the pathology of macular degeneration. Cell Stem Cell. 2021;28(5):846‐862.e8. 33784497 10.1016/j.stem.2021.02.006 PMC8520418

[14] Rivetti di Val Cervo P , Besusso D , Conforti P , Cattaneo E . hiPSCs for predictive modelling of neurodegenerative diseases: dreaming the possible. Nat Rev Neurol. 2021;17(6):381‐392. 33658662 10.1038/s41582-021-00465-0 PMC7928200

[15] Theodoris CV , Zhou P , Liu L , et al. Network‐based screen in iPSC‐derived cells reveals therapeutic candidate for heart valve disease. Science. 2021;371(6530). 10.1126/science.abd0724 PMC7880903 33303684

[16] Kleiman RJ , Engle SJ . Human inducible pluripotent stem cells: realization of initial promise in drug discovery. Cell Stem Cell. 2021;28(9):1507‐1515. 34478628 10.1016/j.stem.2021.08.002

[17] Rohani L , Johnson AA , Naghsh P , Rancourt DE , Ulrich H , Holland H . Concise review: molecular cytogenetics and quality control: clinical guardians for pluripotent stem cells. Stem Cells Transl Med. 2018;7(12):867‐875. 30218497 10.1002/sctm.18-0087 PMC6265634

[18] Yates AG , Pink RC , Erdbrügger U , et al. In sickness and in health: the functional role of extracellular vesicles in physiology and pathology in vivo: part I: health and normal physiology: part I: health and normal physiology. J Extracell Vesicles. 2022;11(1):e12151. 35041249 10.1002/jev2.12151 PMC8765331

[19] Chen Y , Zhu Q , Cheng L , et al. Exosome detection via the ultrafast‐isolation system: EXODUS. Nature Methods. 2021;18(2):212‐218. 33432243 10.1038/s41592-020-01034-x

[20] Crewe C , Funcke JB , Li S , et al. Extracellular vesicle‐based interorgan transport of mitochondria from energetically stressed adipocytes. Cell Metab. 2021;33(9):1853‐1868.e11. 34418352 10.1016/j.cmet.2021.08.002 PMC8429176

[21] Greenhill C . Role for exosomes in insulin sensitivity. Nat Rev Endocrinol. 2021;17:706. 10.1038/s41574-021-00570-6 34545232

[22] Liu YY , Li Y , Wang L , et al. Mesenchymal stem cell‐derived exosomes regulate microglia phenotypes: a promising treatment for acute central nervous system injury. Neural Regen Res. 2023;18(8):1657‐1665. 36751776 10.4103/1673-5374.363819 PMC10154505

[23] Tian Y , Ma L , Gong M , et al. Protein profiling and sizing of extracellular vesicles from colorectal cancer patients via flow cytometry. ACS Nano. 2018;12(1):671‐680. 29300458 10.1021/acsnano.7b07782

[24] Livak KJ , Schmittgen TD . Analysis of relative gene expression data using Real‐Time quantitative PCR and the 2−ΔΔCT method. Methods. 2001;25(4):402‐408. 11846609 10.1006/meth.2001.1262

[25] Nakano‐Kobayashi A , Fukumoto A , Morizane A , et al. Therapeutics potentiating microglial p21‐Nrf2 axis can rescue neurodegeneration caused by neuroinflammation. Sci Adv. 2020;6(46). 10.1126/sciadv.abc1428 PMC7673758 33188020

[26] Ng RC , Jian M , Ma OK , et al. Chronic oral administration of adiporon reverses cognitive impairments and ameliorates neuropathology in an alzheimer's disease mouse model. Mol Psychiatry. 2020. 10.1038/s41380-020-0701-0 32132650

[27] Pape K , Tamouza R , Leboyer M , Zipp F . Immunoneuropsychiatry ‐ novel perspectives on brain disorders. Nat Rev Neurol. 2019;15(6):317‐328. 30988501 10.1038/s41582-019-0174-4

[28] Pröbstel AK , Zhou X , Baumann R , et al. Gut microbiota‐specific IgA(+) B cells traffic to the CNS in active multiple sclerosis. Science Immunology. 2020;5(53). 10.1126/sciimmunol.abc7191 PMC8043673 33219152

[29] Yang Q , Vazquez AL , Cui XT . Long‐term in vivo two‐photon imaging of the neuroinflammatory response to intracortical implants and micro‐vessel disruptions in awake mice. Biomaterials. 2021;276:121060. 34419839 10.1016/j.biomaterials.2021.121060 PMC8409342

[30] Cupovic J , Onder L , Gil‐Cruz C , et al. Central nervous system stromal cells control local CD8(+) T cell responses during Virus‐Induced neuroinflammation. Immunity. 2016;44(3):622‐633. 26921107 10.1016/j.immuni.2015.12.022 PMC7111064

[31] Kim C , Beilina A , Smith N , et al. LRRK2 mediates microglial neurotoxicity via NFATc2 in rodent models of synucleinopathies. Sci Transl Med. 2020;12(565). 10.1126/scitranslmed.aay0399 PMC8100991 33055242

[32] Lopez‐Rodriguez AB , Hennessy E , Murray CL , et al. Acute systemic inflammation exacerbates neuroinflammation in alzheimer's disease: IL‐1beta drives amplified responses in primed astrocytes and neuronal network dysfunction. Alzheimers Dement. 2021. 10.1002/alz.12341 PMC8874214 34080771

[33] Subedi L , Kwon OW , Pak C , et al. N,N‐disubstituted azines attenuate LPS‐mediated neuroinflammation in microglia and neuronal apoptosis via inhibiting MAPK signaling pathways. BMC Neurosci. 2017;18(1):82. 29281977 10.1186/s12868-017-0399-3 PMC5745756

[34] Xu J , Yuan C , Wang G , et al. Urolithins attenuate LPS‐Induced neuroinflammation in BV2Microglia via MAPK, akt, and NF‐κB signaling pathways. J Agricult Food Chem. 2018;66(3):571‐580. 10.1021/acs.jafc.7b03285 29336147

[35] Isaac R , Reis FCG , Ying W , Olefsky JM . Exosomes as mediators of intercellular crosstalk in metabolism. Cell Metab. 2021;33(9):1744‐1762. 34496230 10.1016/j.cmet.2021.08.006 PMC8428804

[36] Lim GB . Exosome‐eluting stents improve vascular remodelling. Nat Rev Cardiol. 2021;18(6):386. 10.1038/s41569-021-00557-w 33888883

[37] Robson A . Exosome‐derived microRNAs improve cardiac function. Nat Rev Cardiol. 2021;18(3):150‐151. 10.1038/s41569-020-00498-w 33349670

[38] Winkle M , El‐Daly SM , Fabbri M , Calin GA . Noncoding RNA therapeutics ‐ challenges and potential solutions. Nat Rev Drug Discovery. 2021;20(8):629‐651. 34145432 10.1038/s41573-021-00219-z PMC8212082

[39] Hou J , Peng W . LIMITing tumours with an immunogenic lncRNA. Nature Cell Biol. 2021;23(5):443‐445. 33958759 10.1038/s41556-021-00682-1

[40] Statello L , Guo CJ , Chen LL , Huarte M . Gene regulation by long non‐coding RNAs and its biological functions. Nat Rev Mol Cell Biol. 2021;22(2):96‐118. 33353982 10.1038/s41580-020-00315-9 PMC7754182

[41] Upadhya R , Madhu LN , Attaluri S , et al. Extracellular vesicles from human iPSC‐derived neural stem cells: miRNA and protein signatures, and anti‐inflammatory and neurogenic properties. J Extracell Vesicles. 2020;9(1):1809064. 32944193 10.1080/20013078.2020.1809064 PMC7480597

[42] Zong L , Huang P , Song Q , Kang Y . Bone marrow mesenchymal stem cells‐secreted exosomal H19 modulates lipopolysaccharides‐stimulated microglial M1/M2 polarization and alleviates inflammation‐mediated neurotoxicity. Am J Transl Res. 2021;13(3):935‐951. 33841631 PMC8014338

[43] Fellner A , Barhum Y , Angel A , et al. Toll‐Like Receptor‐4 inhibitor TAK‐242 attenuates motor dysfunction and spinal cord pathology in an amyotrophic lateral sclerosis mouse model. Int J Mol Sci. 2017;18(8):1666. 28763002 10.3390/ijms18081666 PMC5578056

[44] Hines DJ , Choi HB , Hines RM , Phillips AG , MacVicar BA . Prevention of LPS‐induced microglia activation, cytokine production and sickness behavior with TLR4 receptor interfering peptides. PLoS One. 2013;8(3):e60388. 23555964 10.1371/journal.pone.0060388 PMC3610686

[45] Lester SN , Li K . Toll‐like receptors in antiviral innate immunity. J Mol Biol. 2014;426(6):1246‐1264. 24316048 10.1016/j.jmb.2013.11.024 PMC3943763

